# Injectable conductive hydrogel can reduce pacing threshold and enhance efficacy of cardiac pacemaker

**DOI:** 10.7150/thno.54959

**Published:** 2021-02-06

**Authors:** Zhao An, Jun Wu, Shu-Hong Li, Shanglin Chen, Fang-Lin Lu, Zhi-Yun Xu, Hsing-Wen Sung, Ren-Ke Li

**Affiliations:** 1 Toronto General Hospital Research Institute, Division of Cardiovascular Surgery, University Health Network, Toronto, Ontario, Canada.; 2 Department of Cardiovascular Surgery, Changhai Hospital, Naval Medical University, Shanghai, China.; 3 Department of Chemical Engineering, National Tsing Hua University, Hsinchu, Taiwan (ROC).

**Keywords:** conductive biomaterials, pacing threshold, tissue resistance, pacemaker, cardiac arrhythmia.

## Abstract

**Background:** Pacemaker implantation is currently used in patients with symptomatic bradycardia. Since a pacemaker is a lifetime therapeutic device, its energy consumption contributes to battery exhaustion, along with its voltage stimulation resulting in local fibrosis and greater resistance, which are all detrimental to patients. The possible resolution for those clinical issues is an injection of a conductive hydrogel, poly-3-amino-4-methoxybenzoic acid-gelatin (PAMB-G), to reduce the myocardial threshold voltage for pacemaker stimulation. **Methods:** PAMB-G is synthesized by covalently linking PAMB to gelatin, and its conductivity is measured using two-point resistivity. Rat hearts are injected with gelatin or PAMB-G, and pacing threshold is evaluated using electrocardiogram and cardiac optical mapping. **Results:** PAMB-G conductivity is 13 times greater than in gelatin. The *ex vivo* model shows that PAMB-G significantly enhances cardiac tissue stimulation. Injection of PAMB-G into the stimulating electrode location at the myocardium has a 4 times greater reduction of pacing threshold voltage, compared with electrode-only or gelatin-injected tissues. Multi-electrode array mapping reveals that the cardiac conduction velocity of PAMB-G group is significantly faster than the non- or gelatin-injection groups. PAMB-G also reduces pacing threshold voltage in an adenosine-induced atrial-ventricular block rat model. **Conclusion:** PAMB-G hydrogel reduces cardiac pacing threshold voltage, which is able to enhance pacemaker efficacy.

## Introduction

The first cardiac pacemaker was implanted in a patient with atrioventricular block in 1958 1. Since then, cardiac pacemaker performance has been greatly improved by advanced intelligent designs and longevity, which has prevented sudden cardiac death and improved quality of life for patients with cardiac arrhythmia. However, even with low-energy electronics and improved battery life, pacemaker longevity is still significantly impacted by the need for constant electrostimulation, and some patients need to replace a battery-exhausted pacemaker 2. Regional inflammation results from the response of the myocardial tissue to the electrodes, leading to tissue damage and fibrotic tissue formation. The local stimulation of the pacemaker leads often induces further regional fibrosis 3, which will require greater stimulation to maintain cardiac rhythm. Therefore, pacemaker threshold is an important parameter for measuring energy consumption and tissue damage 4-6. Alteration of the interface between the myocardium and the stimulator, in order to reduce the threshold, is helpful for pacemaker efficiency.

A number of technologies have been developed to improve pacemaker function. Porous electrode tips are developed to reduce pacing thresholds 7. Steroid-eluting tips reduce the inflammatory response and decrease local fibrosis, resulting in lower stimulation thresholds 8. Carbon tip electrodes can also reduce the pacing threshold 9. These modifications are effective, but battery life is still limited and additional techniques are required to further reduce myocardial impedance and lower the threshold for pacemaker stimulation.

Conductive materials are polymers characterized by electric conductivity 10. Previously, we created a conductive polymer by grafting pyrrole to the clinically-tested biomaterial, chitosan, to create a polypyrrole (PPy)-chitosan hydrogel. We found this injectable semi-conductive hydrogel can electrically connect contracting cells at a distance. *In vivo* studies revealed that injection of PPy-chitosan after myocardial infarction (MI) decreased the QRS interval and increased the transverse activation velocity, suggesting improved electric conduction 11. Subsequently, we further confirmed that PPy-chitosan established synchronous contraction in two distinct clusters of spontaneously-beating CMs. Intra-myocardial PPy-chitosan injection into the cryo-ablation-induced injured region improved electrical impulse propagation across the scarred tissue 12. However, chitosan, the backbone used to graft PPy, induced cellular infiltration and inflammation at the injected site in long-term studies. Furthermore, unlike collagen and gelatin, which are both components of the extracellular matrix, chitosan is not an optimal material for cellular attachment. Based on these concerns, in the present study, we replaced chitosan with gelatin.

Gelatin is a natural biocompatible protein with good mechanical properties 13. While it is a component of the myocardial extracellular matrix, it is not conductive. In the present study, we conjugated a conductive material, 3-amino-4-methoxybenzoic acid (AMB), onto the side chains of gelatin to generate a conductive biomaterial, poly-AMB-gelatin (PAMB-G). We investigated the effect of PAMB-G on cardiac pacing by injecting it into the myocardium to change the electrode-tissue interface, building on our previous report regarding the application of conductive biomaterial into injured myocardium 14. In the current study, we provide novel insights on the occurrence of pacemaker threshold change upon biomaterial injection, revealing that PAMB-G reduced cardiac pacing threshold voltage and enhanced the efficacy of the cardiac pacemaker.

## Methods

### PAMB-G hydrogel synthesis

Gelatin powder (4 g, G2500, Sigma) was dissolved in 10 mL deionized distilled water under mechanical stirring, followed by adding 0.4 g 3-amino-4-methoxybenzoic acid (AMB) powder (B20669, Alfa Aesar). After the AMB was totally dissolved, 0.546 g ammonium persulfate (APS) was added into the solution to polymerize the AMB, and link the AMB polymer to the amino stubs of the gelatin, to form a PAMB-G solution. The polymerization reaction was maintained for 24 h at 50 °C in a water bath. The mixture was then dialyzed (9201140, Spectrum laboratories, New Zealand) at 50 °C for 10 h. After dialysis, freeze drying (Labconco, MO) was used to eliminate the extra water, and the mixture was re-dissolved in 10 mL PBS at 60 °C, pH adjusted to approximately 7.0 with NaOH, then stored at 4 °C. Before usage, 600 µL mixture was cross-linked with 4 µL N-(3-Dimethylaminopropyl)-N'-ethylcarbodiimide hydrochloride (EDC, 22980, Thermo Fisher, MA) and 2 µL N-Hydroxysuccinimide (NHS, 130672, Sigma-Aldrich, MO) for 1 min to form a PAMB-G hydrogel.

### Assays for the electrical properties of PAMB-G hydrogel

A two-point probe resistivity apparatus (HF2IS, Zurich Instruments, Switzerland) was used to measure biomaterial resistance at room temperature. The probes were placed on gelatin or PAMB-G hydrogel at an interval of 1.5 cm. The conductivity (measured in S/cm) was calculated as 1/(2πDR), where D is the distance between probes (cm) and R=V/I, in which I is the supplied current (mA) and V is the corresponding voltage (mV).

### Strength-duration curve, pulse width and voltage testing in Langendorff-perfused rat heart evaluated by ECG

We began the stimulus duration at 10 milliseconds (ms) and 0.5 V/6 Hz, and used the knob on the SD9 stimulator to slowly increase the voltage until the capture (pacing) appeared. The resulting value obtained was the rheobase voltage. Next, we slowly decreased the stimulus duration until the pacing disappeared. The first step was then repeated, in which the stimulus voltage was increased again until the pacing re-appeared. The resulting new stimulus voltage and stimulus duration from the stimulator was recorded. This procedure was repeated until we had 10 different pairs of values. Using this data, the strength-duration curve was plotted. We next fixed the voltage at 3 V (twice the Rheobase value) with 6 Hz frequency, and the pulse width was incrementally increased from 1 ms until capture was successful. We found that no pacing was generated under 3 V and 6 Hz with 1, 2 and 3 ms pulse width electrode-only pacing.

### Measurement of electrode-tissue interface conduction velocity *in vitro*

Conduction velocity of the electrode-tissue interface was measured *in vitro* using adult rat healthy atrial myocardium. The tissue was linked to the stimulation electrodes via gelatin on the anode, and gelatin or PAMB-G on the cathode (Figure 2A-D), respectively. A 3-lead electrocardiograph recorder (Power Lab, AD Instruments, CO) was used to detect myocardial field potentials and a multi-electrode array (MEA, Multichannel Systems Reutlingen, Germany) was used to detect conduction velocity. Stimulation from 20-100 mV for electrocardiogram (ECG) recording and 300-1000 mV for MEA recording was provided with a stimulator (STG 4002, Multichannel Systems Reutlingen, Germany), and all stimulations were at 4 Hz with 4 ms duration. For MEA, the recoding area is 1.5 x 1.5 mm^2^. The conduction velocity was evaluated by Cardio 2D software (Multichannel Systems Reutlingen, Germany), which allows for mapping cardiac excitation on microelectrode arrays, local activation times and delay between electrodes (s). Each electrode is 300 µm apart. The conduction velocity of the action potential is determined by measuring the distance of electrical current traveled (in cm), divided by the time (s) as follows: Conduction velocity = distance (cm)/time (s).

### Pacing threshold voltage measurement in Langendorff-perfused rat heart

All experimental protocols were approved by the Animal Research Centre of the University Health Network and conformed to the Guide for the Care and Use of Laboratory Animals (NIH, 8th Edition, 2011). Sprague Dawley (SD) rats weighing 235-250 g were used in this study. To measure the threshold voltage, hearts were rapidly explanted and cannulated using a blunted 16G needle via the aortic root on ice. The heart was then retrograde-perfused with Krebs-Henseleit (K-H) solution (117 mM NaCl, 24 mM NaHCO_3_, 11.5 mM dextrose, 3.3 mM KCl, 1.25 mM CaCl_2_, 1.2 mM MgSO_4_, 1.2 mM KH_2_PO_4_ equilibrated with 5% CO_2_/95% O_2_ gas) at 37 °C and 10 mL/min. To prevent motion noise, excitation-contraction coupling was blocked with 2, 3-butanedione monoxime (1 mg/mL, B-0753, Sigma-Aldrich, MO). ECG was used to detect cardiac electrical activity and a stimulator (SD9, Grass, Canada) was used to stimulate the heart. Under K-H buffer perfusion, 20 µL of PAMB-G hydrogel was injected into the myocardium at the ventricular apex. The cathode was then inserted into the PAMB-G area and an anode electrode was inserted into the K-H solution about 1.5 cm away from the cathode electrode. As the SD9 stimulator is only equipped with voltage output, we used different voltages to determine the pacing threshold voltage. Stimulation was started at 0.5 V and increased in increments of 0.1 V until ventricular capture was achieved. The lowest value needed to induce a 100% pacing rhythm was recorded as the pacing threshold voltage. In each group, 5 V stimulation was performed and the ECG monitored for electrophysiological analysis. Direct electrode pacing without injection of material and pacing stimulation following gelatin injection served as controls. All stimulations were 6 Hz with 4 ms duration. Pacing energy consumption was calculated according to the equation of E = (U2/R) x T. E is the energy consumption, U the pacing voltage, R the impedance and T the duration time.

### Whole-heart optical mapping

A Langendorff perfusion procedure was performed as described in the section above (Pacing Threshold Voltage Measurement). Five minutes after cardiac recovery with spontaneous beating, the heart was perfused with the voltage-sensitive dye 4-(2-(6-(dibutylamino)-2-naphthalenyl) ethenyl)-1-(3-sulfopropyl)-pyridinium (di-4 ANEPPS; D1199, Invitrogen), dissolved in K-H solution (25 µM) at a rate of 5 mL/min for 6 min. After administration of the dye, PAMB-G hydrogel was injected and electrodes were inserted using the same method described above. 0.5 V and 5 V stimulation was used for the stimulator and the optical mapping data were recorded with a camera (Evolve 128, Photometrics, AZ). Custom made software based on Matlab (MathWorks, MA) was used for data analysis of the optical mapping signals 15. [15]Direct electrode pacing and gelatin injection pacing served as controls. All stimulations were carried out at 6 Hz with 4 ms duration.

### Rat atrioventricular block model

Adenosine (AD; 519 987, Boehringer Mannheim, Germany) was used to induce rat atrioventricular (AV) block. After a median sternotomy, 150 µL AD (10 mg/mL) was rapidly injected via the inferior vena cava to induce AV block, and the time of the block was recorded. The AD dose was adjusted to maintain AV block duration of 120 s 16, 17.

### *In vivo* pacing threshold voltage measurement

Rats were mechanically ventilated and anaesthetized with 2-2.5% isoflurane, and conventional surface ECG was used to monitor and record heart rhythm. Median sternotomy was performed and after adequate heart exposure, 20 µL PAMB-G hydrogel was injected into the right ventricle wall at the apex. After PAMB-G injection, 0.15 mL adenosine (10 mg/mL) was rapidly injected via the inferior vena cava to induce AV block. A cathode was then inserted into the PAMB-G area and an anode was inserted subcutaneously on the left side of the sternum. Stimulation procedures were the same as described for the Langendorff-perfused rat heart model, and were performed 10-15 s after adenosine injection. Pacing threshold voltage values were recorded, and ECGs from 0.5 to 5 V stimulation in each group were also recorded for electrophysiological analysis. The ECGs were also continuously recorded until the hearts returned to sinus rhythm. Direct electrode pacing and gelatin injection pacing served as controls. All stimulations were carried out at 6 Hz with 4 ms duration.

### Statistical analysis

Data are expressed as mean ± standard deviation. Analyses were performed using GraphPad Prism software (v. 7.0), with the critical α-level set at P < 0.05. Conduction velocity and field potential amplitude were analyzed by two-way analysis of variance (ANOVA), followed by Bonferroni *post-hoc* tests. Two-sided student's t-tests were used for comparisons of means between two groups. Comparisons of means among three or more groups were performed using one-way ANOVA. When the ANOVA F-values were significant, differences between groups were determined using Tukey's *post-hoc* tests.

## Results

### PAMB-G hydrogel synthesis and characterization

**Figure 1:**
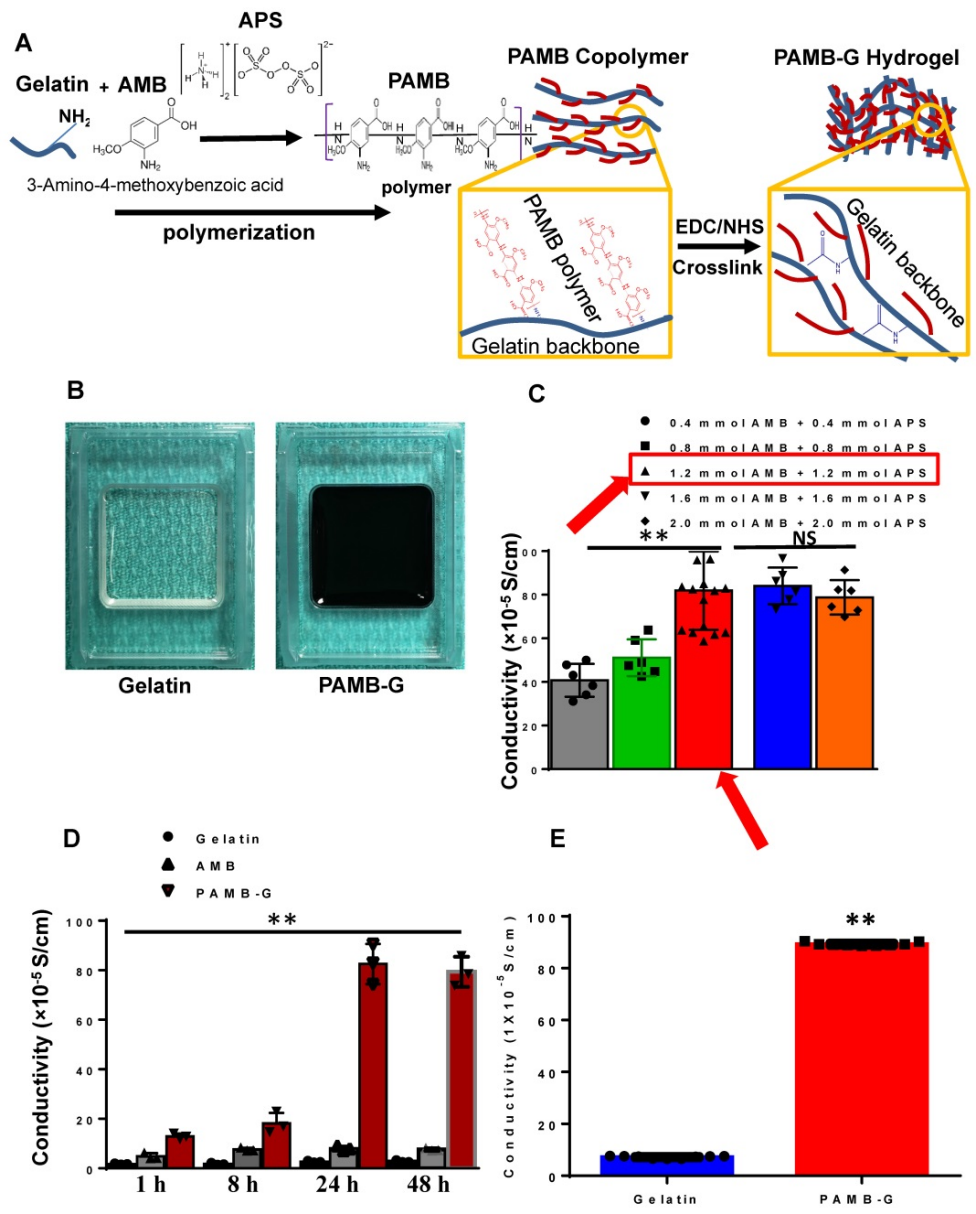
** PAMB-G synthesis and electrical characteristics. (A)** Schematic diagram showing that gelatin and 3-amino-4-methoxybenzoic acid (AMB) monomers were polymerized under ammonium persulfate (APS), followed by cross-linking with N-(3-dimethylaminopropyl)-N'-ethylcarbodiimide hydrochloride (EDC) and N-hydroxysuccinimide (NHS) to form PAMB-gelatin (PAMB-G) hydrogel. **(B)** Gelatin (left) and PAMB-G (right) hydrogel under room temperature. **(C)** Optimal concentration for the polymerization of AMB and conjugation to gelatin. AMB monomers were conjugated to a gelatin (G) in the presence of APS to create a poly-AMB (PAMB)-G solution. The optimal concentration in terms of maximizing the conductive capacity was 1.2 mM AMB + 1.2 mM APS. **(D)** Optimal reaction time for the polymerization of AMB and conjugation to gelatin. The optimal reaction time in terms of maximizing the conductive capacity was determined to be 24 h. **(E)** PAMB-G showed significantly higher conductivity than gelatin. n = 9/group for C and E, 3 for D, **P < 0.01. Conductivity between gelatin and PAMB-G analysis used two-sided student's t-test. Remaining data analysis used one-way analysis of variance (ANOVA) followed by Tukey's *post- hoc* tests. Data shown as mean ± SD.

As shown in Figure 1A, AMB monomers were polymerized to polymers and connected to the amides on the gelatin molecule to construct PAMB-G. We used ammonium persulfate (APS) to catalyze this reaction. The PAMB-G solution was cross-linked with EDC and NHS to form a PAMB-G hydrogel (Figure 1B). The conductive measurement showed that the optimal concentration is 1.2 mM AMB with 1.2 mM APS (Figure 1C, red arrows). We evaluated the reaction time for optimal synthesis of the conductive biomaterial by measuring the material conductivity at 1, 8, 24 and 48 h, and found that the maximum conductivity was present at 24 h. Afterwards, there is only a slightly lower conductivity level at 48 h (no statistically-significant difference, compared with 24 h). Based on these findings, we selected 24 h as the optimal reaction time for synthesizing the biomaterial (Figure 1D). The PAMB-G overall had significantly enhanced conductivity compared with gelatin (Figure 1E).

### PAMB-G hydrogel increased *ex vivo* cardiac field potential amplitude and conduction velocity

**Figure 2:**
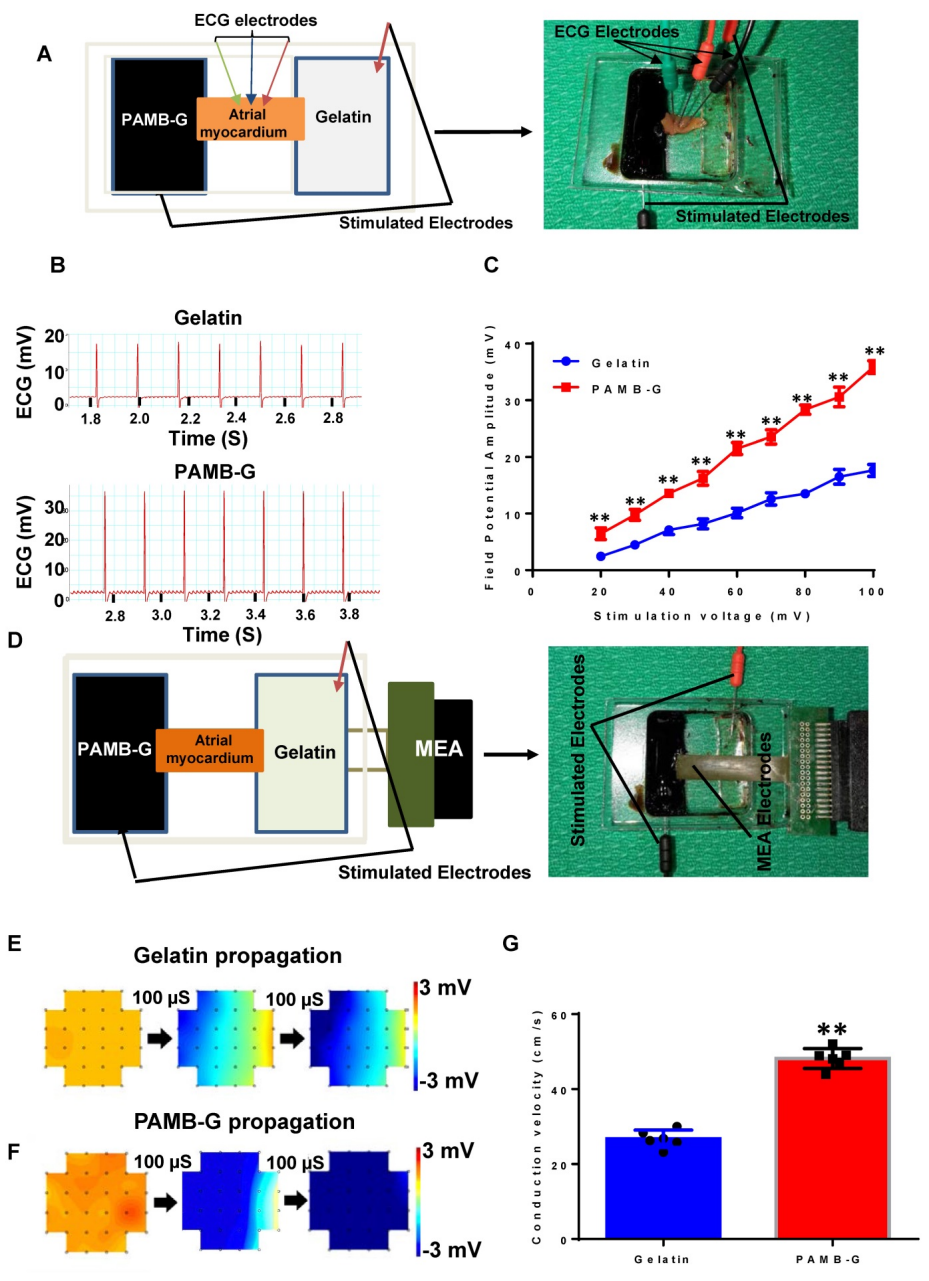
**PAMB-G hydrogel increased cardiac field potential amplitude and conduction velocity evaluated by multi-electrode array (MEA). (A)** Schematic drawing (left) and photograph of the assay. **(B)** Representative isolated atrial myocardium ECGs from gelatin and PAMB-G at 100 mV stimulation voltage. **(C)** Field potential amplitude was significantly higher in PAMB-G than gelatin under 20-100 mV stimulation. **(D)** Schematic drawing (left) and photograph of the MEA array used to measure tissue conduction. **(E-F)** Representative MEA images illustrate the propagation of electrical current across the biomaterial-injected regions at 500 mV stimulation voltage. **(G)** Conduction velocity was significantly faster in PAMB-G than gelatin under 500 mV stimulation. n = 6/group for C and G, **P < 0.01. Data analysis used two-way analysis of variance (ANOVA) followed by Bonferroni *post-hoc* tests for C and two-sided student's t-test for G. Data shown as mean ± SD.

We developed an *ex vivo* model to simulate the electrode-tissue interface with isolated atrial myocardium to compare the conduction velocity between PAMB-G and gelatin. We measured the field potential amplitude of the isolated atrial tissue under different stimulation voltages and monitored using ECG (Figure 2A, and B showed that the stimulation voltages were 100 mV for both gelatin and PAMB-G). Myocardial field potential amplitude was significantly greater in PAMB-G than gelatin (Figure 2C). We then detected the conduction velocity of the isolated atrial myocardium at different stimulation voltages with MEA monitoring (Figure 2D). The electrical current propagated across the whole inter-electrode (stimulating electrodes) region within 200 µs in PAMB-G, while in the gelatin group, the electrical current only propagated across half of the inter-electrode region (blue colors in Figure 2E and 2F, stimulation voltages are 500 mV for both gelatin and PAMB-G). Compared to the gelatin group, the conduction velocity in the PAMB-G group is significantly faster when the stimulation voltage is at 500 mV (Figure 2G). These data suggest that we have successfully synthesized a conductive PAMB-G hydrogel, which showed significantly higher conductivity, improved electrical propagation, and enhanced conduction velocity compared to gelatin control.

### Strength-duration curve, pulse width and voltage testing in Langendorff-perfused rat heart evaluated by ECG

**Figure 3:**
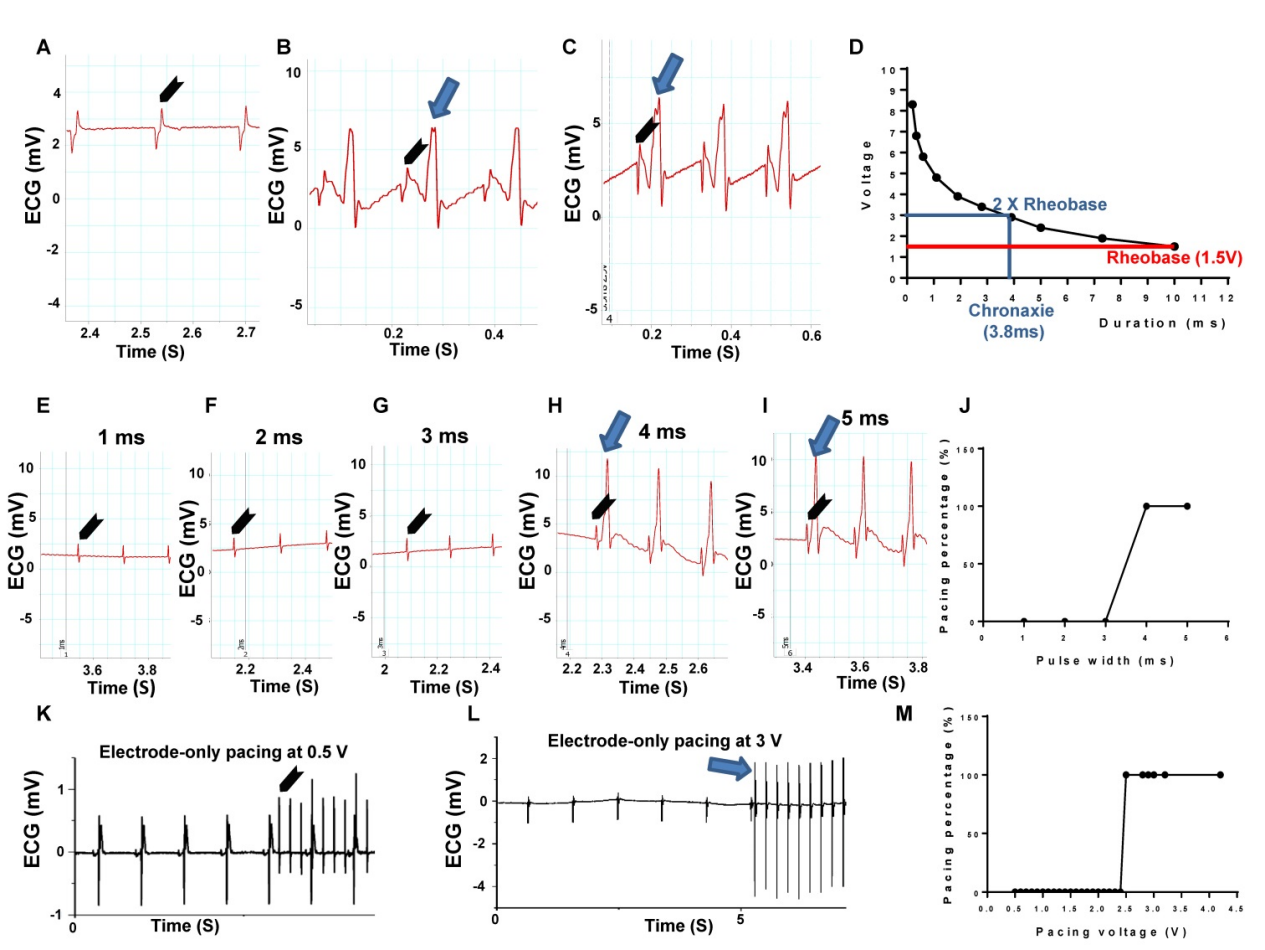
** Strength-duration curve, pulse width and voltage testing in a Langendorff-perfused rat heart evaluated by ECG. (A)** Representative ECG trace under 1.2 V, 6 Hz with 10 ms pulse width electrode-only pacing. No pacing was generated. **(B)** Representative ECG traces showed pacing rhythm when pacing voltage was increased to 1.5 V with 10 ms width at 6 Hz, which was the rheobase strength. **(C)** Representative ECG traces showed pacing rhythm under pacing voltage at 2.9 V with 3.9 ms width at 6 Hz. **(D)** Plotted strength-duration curve. The chronaxie voltage was twice that of the Rheobase, yielding a 3.8 ms duration. **(E-G)** Representative ECG traces under 3 V at 6 Hz, with 1 to 3 ms pulse width electrode-only pacing. No pacing was generated. **(H-I)** Representative ECG traces showed a pacing rhythm when pacing pulse width increased to 4 or 5 ms, under 3 V at 6 Hz. **(J)** Plotted curve of pulse width duration from 1 to 5 ms (n = 4). **(K)** Representative ECG traces with 4 ms width and 6 Hz, under 0.5 V pulse voltage electrode-only pacing. No pacing was generated. **(L)** Representative ECG traces showed a pacing rhythm when pacing voltage increased to 3 V, with 4 ms width at 6 Hz. **(M)** Plotted curve of pulse voltage from 0.5 to 4.2 V. n = 6 for all tests except J. Arrow head: stimulation; Arrow: pacing**.**

To evaluate myocardial stimulation threshold, we first determined rheobase and chronaxie values, which are points defined on a strength-duration curve for stimulus of an excitable tissue. Rheobase is the lowest intensity with indefinite pulse duration that results in a muscle being stimulated. Chronaxie is the minimum time required to stimulate a muscle, using an electric current double the strength of the rheobase value. We initiated the stimulus duration at 10 ms, 0.5 V/6 Hz and slowly increased the voltage until the capture (pacing) appeared. The value given was the rheobase voltage. Using 10 different pairs of values, the strength-duration curve was plotted (Figure 3D). Figure 3A is a representative ECG trace under 1.2 V, 6 Hz with 10 ms pulse width electrode-only pacing. No pacing was generated. Figure 3B demonstrates a representative ECG trace, depicting the pacing rhythm when the pacing voltage was increased to the rheobase strength of 1.5 V with 10 ms width at 6 Hz. Figure 3C shows a representative ECG trace, depicting pacing rhythm close to the chronaxie value, which was twice the strength of the rheobase voltage at 2.9 V, with 3.9 ms width at 6 Hz. The corresponding strength-duration curve yielded a width of 3.8 ms under the chronaxie value. Figure 3D was a plotted strength-duration curve. We then evaluated different pacing pulse widths, in which the voltage was fixed at 3 V (twice the Rheobase value) and 6 Hz. The pulse width was incrementally increased from 1 ms until capture was successful. Figures 3E-G showed the representative ECG traces under 3 V and 6 Hz, with 1 to 3 ms pulse width pacing. Under such parameters, no pacing was generated. Figures 3H-I were the representative ECG traces showing the pacing rhythm when pacing pulse width increased to 4 or 5 ms under 3 V at 6 Hz. Figure 3J was a plotted curve of pulse width duration, in which 4 ms pulse width was chosen as an optimal pacing pulse width for our experiments 18.

Finally, we tested the pulse voltage threshold in electrode-only pacing. The pulse width is fixed at 4 ms and 6 Hz, while the pulse voltage was incrementally increased from 0.5 V until capture was successful. Figure 3K was a representative ECG trace for the electrode-only heart, paced at 4 ms width and 6 Hz, with 0.5 V pulse voltage. The ECG analysis showed complete separation of stimulated wave and heart rhythms, suggesting unsuccessful pacing. Figure 3L was also an electrode-only heart ECG trace, showing synchronization between the pacing rhythm and heartbeat when pacing voltage increased to 3 V, with 4 ms width at 6 Hz. Figure 3M was a plotted curve of pulse voltage from 0.5 to 4.2 V, in which the average of 3 V pulse voltage was chosen as an optimal pacing voltage.

These research data suggested a technique to evaluate pacing threshold with pulse width at 4 ms and 6 Hz, and incrementally increased the pulse voltage from 0.5 V until capture was successful. We found that the average of 3 V pulse voltage was an optimal pacing voltage in normal myocardium.

### PAMB-G hydrogel decreased cardiac pacing threshold voltage in adult rat heart

**Figure 4:**
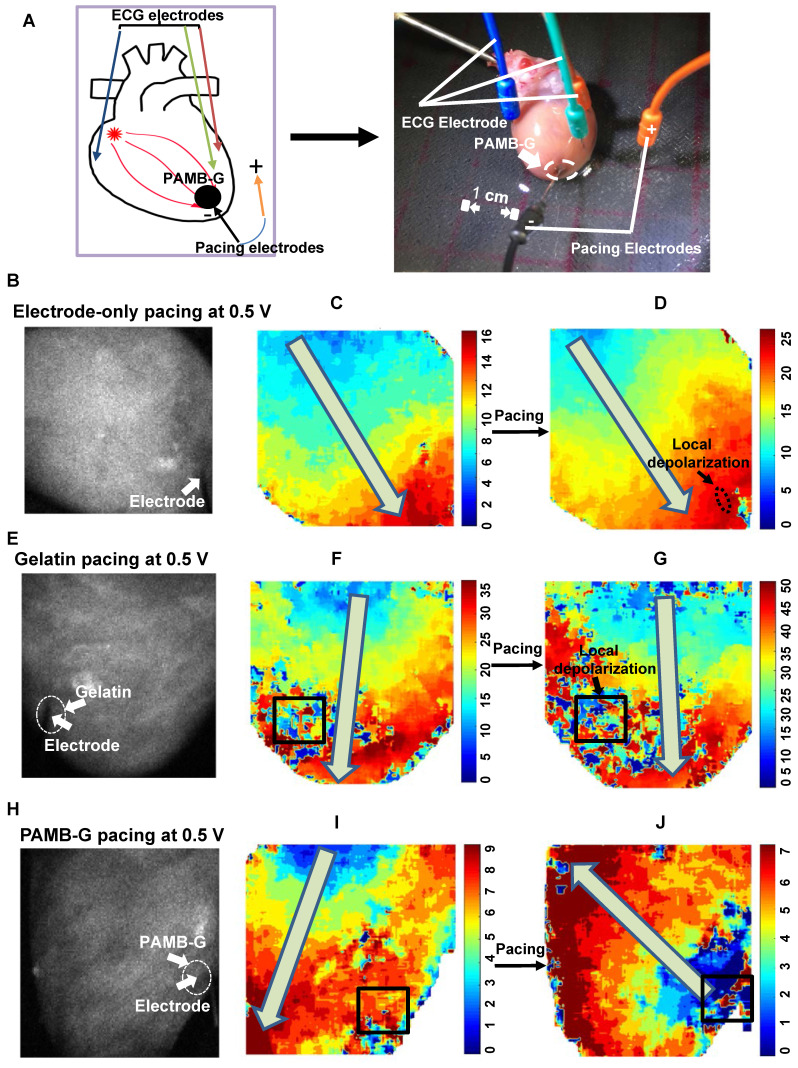
** PAMB-G decreased heart pacing threshold voltage in the Langendorff-perfused rat heart evaluated by optical mapping. (A)** Schematic drawing (left) and photograph of the Langendorff-perfused rat heart pacing model. **(B-D)** Representative optical mapping images in electrode-only pacing at 0.5 V. The electrode was inserted near the apex of the heart (white arrow). Stimulation did not pace the heart and only induced a local depolarization (D, black arrow). Activation orientation from base to apex was indicated by grey arrows. **(E-G)** Representative optical mapping images in gelatin pacing at 0.5 V. Square line boxes highlight the injection areas. Stimulation did not pace the heart and the gelatin-injection area showed local depolarization only (G, black arrow). Activation orientation from base to apex was indicated by grey arrows. **(H-J)** Representative optical mapping images in PAMB-G group pacing at 0.5 V. Stimulation paced the heart successfully by changing the activation orientation from apex to base (J, grey arrow).

To evaluate the effect of PAMB-G on reduction of cardiac pacing threshold voltage, we perfused adult rat hearts using a Langendorff apparatus, beating in sinus rhythm (Figure 4A). The pacing probe was placed at the left ventricle and 0.5 V stimulation was used. Figures 4B, 4E and 4H were representative black and white raw optical mapping images while figures 4C, 4F and 4I were representative pseudo color optical mapping images. A square line box indicated the material injection area for each heart image (Fig. 4F-G, I-J). When not stimulated, the heart conduction is controlled by SA node. Depolarization without pacing occurs from the base of the heart to the apex, as indicated by the arrows in Figures 4F and 4I. Optical mapping showed a small local depolarization area in electrode-only and gelatin-injected areas (small black arrows, Figure 4D and 4G, respectively) without changing the direction of electrical current propagation (large grey arrows, Figure 4D and 4G, as well as Movie S1, 2, respectively), upon stimulation. It should be noted, though, that the gelatin changed the local conduction pathway, resulting in non-smooth regional conduction where the regional electrical current diminished over time (Square box in Figure 4F). However, in the PAMB-G group, 0.5 V stimulation was enough to change the direction of electrical current propagation, from apex to the base, thus resulting in global depolarization (Figure 4H to 4J, grey arrow, and Movie S3). Optical mapping also showed an ectopic pacemaker at the PAMB-G injection area (blue area, Figure 4J).

**Figure 5:**
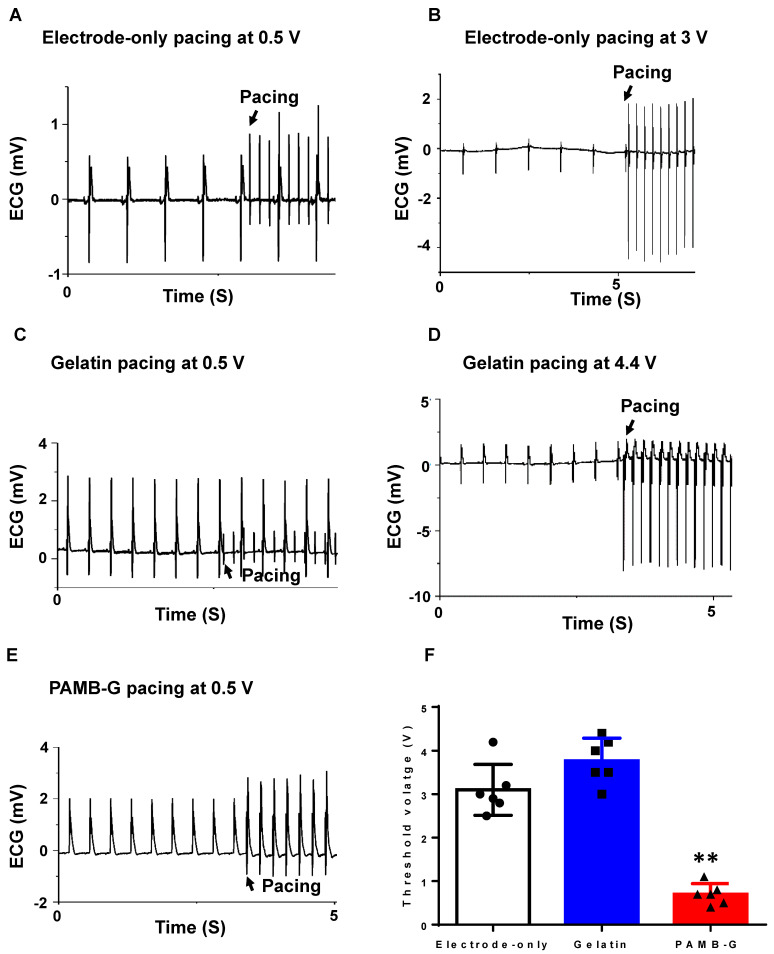
**PAMB-G decreased heart pacing threshold voltage in the Langendorff-perfused rat heart evaluated by ECG. (A)** Representative ECG traces under 0.5 V electrode-only pacing. Stimulation and cardiac rhythm were mutually independent. **(B)** Representative ECG traces showed a pacing rhythm when pacing voltage increased to 3 V. **(C)** Representative ECG traces under 0.5 V gelatin pacing. Stimulation and cardiac rhythm were mutually independent. **(D)** Representative ECG traces showed a pacing rhythm when pacing voltage increased to 4.4 V. **(E)** Representative ECG traces under 0.5 V PAMB-G pacing. Stimulation successfully induced whole heart depolarization and the heart was under the pacing rhythm. **(F)** PAMB-G pacing had a significantly lower threshold voltage compared with electrode-only or gelatin pacing. n = 6/group for F, **P < 0.01 compared with electrode-only and gelatin. Data analysis used one-way analysis of variance (ANOVA) followed by Tukey's *post- hoc* tests. Data shown as mean ± SD.

The ECG analysis showed that the electrode-only heart group had a completely separate stimulation wave and heart rhythm (Figure 5A) when pacing at 0.5 V, and can only be paced at ~3 V (Figure 5B). In the gelatin group at 0.5 V stimulation, the ECG also showed completely separate stimulation waves and heart rhythm tracings (Figure 5C), and can only be paced at 4.4 V (Figure 5D). However, in the PAMB-G group, 0.5 V stimulation was high enough to change the rhythm from autonomous cardiac rhythm to the pacing rhythm (Figure 5E). These data suggest that 0.5 V stimulation into the conductive biomaterial of PAMB-G resulted in cardiac depolarization by reducing the pacing threshold. To evaluate the lowest voltage needed to induce heart depolarization in the three groups of hearts, we increased the stimulating voltage to identify the threshold for heart pacing (synchronization of pacing and autonomous heart rates). PAMB-G had the lowest cardiac pacing threshold voltage compared with electrode-only or gelatin pacing (Figure 5F).

### PAMB-G hydrogel reduced pacing threshold voltage and enhanced efficacy of cardiac pacemaker *in vivo*

**Figure 6:**
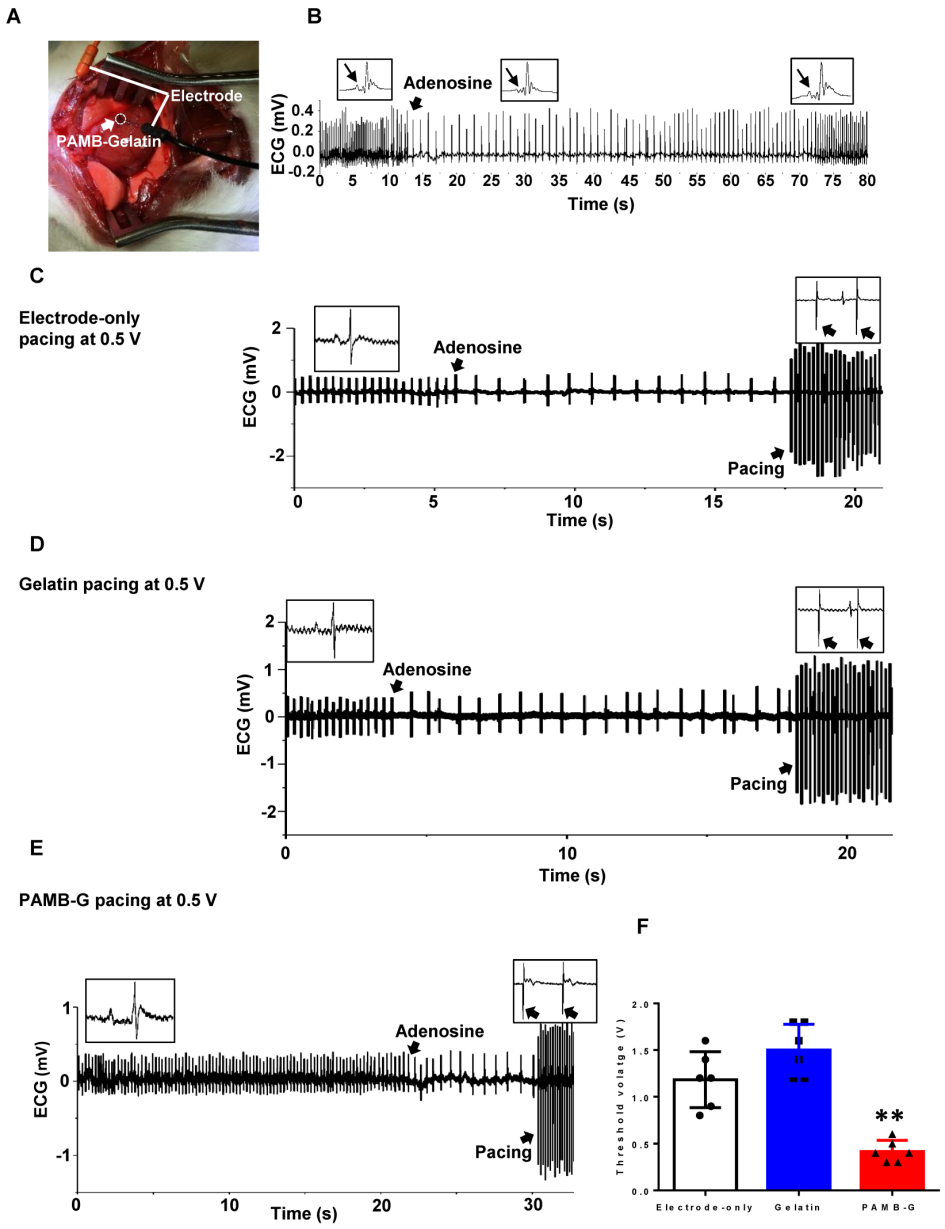
** PAMB-G decreased heart pacing threshold voltage *in vivo*. (A)** PAMB-G *in vivo* pacing model. The cathode was inserted in the PAMB-G area near the apex of the heart and the anode was inserted in the left side of the sternum subcutaneously. **(B)** Representative ECG traces showed that adenosine (AD) injection inhibited sinoatrial node electrical activity, inducing an inverted P wave in the ECG trace. Sinus rhythm spontaneously recovered 60 s after AD injection. Enlarged ECG traces were shown in boxes, and P wave indicated by arrows. Representative ECG traces under electrode-only **(C)**, gelatin **(D)** and PAMB-G **(E)** at 0.5 V pacing. Stimulation and cardiac rhythm were mutually independent under electrode-only and gelatin pacing (black arrows in insert boxes), while PAMB-G pacing successfully induced whole heart depolarization, and the heart was in a pacing rhythm (black arrows in insert boxes). **(F)** PAMB-G pacing showed significantly lower threshold voltage compared with electrode-only or gelatin pacing. n = 6/group for F, **P < 0.01 compared with electrode-only and gelatin. Data analysis used one-way analysis of variance (ANOVA) followed by Tukey's *post- hoc* tests. Data shown as mean ± SD.

To evaluate pacing characteristics, we injected AD through the inferior vena cava to decrease heart rate *in vivo* (Figure 6A). After AD injection, the sinus node was suppressed, with a reversed P wave visible on the ECG along with decreased heart rate (Figure 6B). Representative ECGs showed that electrode stimulation using 0.5 V/6 Hz and 4 ms duration in the electrode-only and gelatin groups resulted in totally separated pacing tracings along with an autonomous rhythm (Figure 6C and 6D). In the PAMB-G group, heart rhythm changed to a totally paced rhythm with an increased heart rate (Figure 6E). The pacing threshold voltage in the PAMB-G group was significantly decreased compared to the electrode-only and gelatin groups (Figure 6F). These data suggest that PAMB-G hydrogel injection reduced the pacing threshold voltage and enhanced efficacy of cardiac pacemaker *in vivo*, which corroborated our findings from the *ex vivo* Langendorff-perfused rat heart model.

To explore the mechanism of PAMB-G affecting electrode-tissue interface, we established an equivalent circuit model (Figure S1A). To quantify the difference in electrical parameters between conductive (PAMB-G) and non-conductive (gelatin) biomaterials, impedance measurements were carried out. The frequency response of each biomaterial was measured from 100 Hz-100 kHz with an applied voltage of 100 mV. We found that the impedance of electrode-only and gelatin groups were higher than that of PAMB-G at 7 kHz frequency (Figure S1B). Power spectrum analysis showed that the energy (voltage) that remained after passing through PAMB-G at 1 Hz was ~40% greater than that passing through electrode-only or gelatin (Figure S1C). Additionally, the pacing energy consumption was significantly decreased in PAMB-G group compared with electrode-only and gelatin groups (Figure S1D). These results implied that PAMB-G helped to reduce the impact of the electrode-tissue impedance and facilitated the electrical current energy transfer.

Taken together, our experiment results showed that PAMB-G reduced cardiac pacing threshold voltage and improved pacing electrophysiological performance by providing a higher efficiency of electrode-tissue interface energy transfer.

## Discussion

In the present study, we synthesized a new conductive biomaterial, PAMB-G hydrogel and demonstrated that it reduced cardiac pacing threshold voltage after injection into the area around the electrode-myocardial interface. Our data provide mechanistic insight into the effect of this conductive biomaterial and demonstrate its potential for reducing pacemaker energy consumption.

When we generated the conductive biomaterial, we determined that PAMB could not be used for direct injection into myocardial tissue because it is difficult to transform into a gel and has poor biocompatibility. To enhance biocompatibility and increase viscosity, we conjugated PAMB on gelatin, a natural protein derived from collagen. Previous studies have used gelatin-based macromolecules in bioactive molecules to control release 19. Octacalcium phosphate and gelatin composite have been used in bone regeneration 20, and a polycitrate-gelatin hybrid polymer has been used in tissue regeneration because of its better biocompatibility and mechanical properties 21. Additionally, it has been used as a scaffold for cell growth and chemical modification because of its natural structure 22. In the current study, we conjugated PAMB onto gelatin, which resulted in better biocompatibility. We also evaluated the optimal concentration and reaction time in terms of maximizing the conductivity of the resulting biomaterial.

The selection of conductive polymers is a key factor for their success in biomedical applications. Due to the necessity of conjugating the conductive polymers onto the biomaterial, such as gelatin or chitosan, in order to yield a conductive material, we focused on molecules able to be grafted onto the gelatin, or other biological molecules. In our first generation of conductive biomaterial, we found that PPY is able to be grafted onto the chitosan, and this resulting combination was used for the biological application. Since ferric chloride is a necessary component for doping the PPY, albeit with the risk of inducing cardiac damage, we decided that even though PPY-Chitosan is able to restore cardiac conduction, it was preferable to generate a new conductive biomaterial using PAMB. The major advantage of using PAMB is its self-doping capacity being able to contribute to its conductive capacity, which differentiates this material from most conductive polymers, whose conductive capacity is doping reagent-dependent. Furthermore, PAMB is able to be conjugated onto gelatin, which is a more biocompatible material compared to chitosan. Therefore, we selected as our conductive biomaterial PAMB conjugated to gelatin.

With respect to other popular conductive polymers, such as polyaniline and poly(3,4-ethylenedioxythiophene) polystyrene sulfonate (PEDOT:PSS), polyaniline only demonstrates its conductive properties at pH < 4.0, making it unsuitable for biological applications. As for PEDOT:PSS, it consists of a polymer mixture of two ionomers. This conductive polymer was not selected owing to concerns regarding molecule conjugation, in that an uneven ratio of the ionomers may make it difficult to control the conductive property of the polymer.

The pacemaker is widely used in the treatment of myocardial conductive dysfunction, such as AV-block and heart failure with intraventricular delayed conduction 5. Pacemaker battery life becomes a functional issue when the initiation of myocardial depolarization must overcome increased impedance due to local fibrosis. Scientists have identified multiple factors affecting battery life and actively investigated for the solutions. Several new techniques have been developed to address this issue, such as reducing the electrode surface area 23, adopting a microporous structure in the cathode electrode 7, as well as the use of new materials 24-26 and steroid-eluting leads to inhibit local fibrosis 8. Application of these techniques has reduced pacemaker threshold voltage 23, 27, 28 and prolonged pacemaker battery life 29. The drawback, though, is that the energy consumption of current pacemakers is still high, and most patients need a second operation to replace an exhausted battery 30. However, by using PAMB-G, we have increased tissue field potentials and conduction velocity, as well as lowering the tissue impedance and cardiac energy consumption required for the pacemaker. Therefore, the stimulation threshold of the pacemaker was lowered.

We aimed to find the minimum voltage needed to pace heart in each group. We first determined the rheobase and chronaxie values for electrode pacing in a Langendorff-perfused rat heart. Afterwards, different pacing pulse widths from 1 to 5 ms were tested and evaluated by ECG. We found that 4 ms pulse width was the optimal pacing pulse width, which was also supported with a previous experiment using rabbit heart, where 4 ms pulse width was also chosen as an optimal pulse width in the Langendorff-perfused rabbit heart for pacing threshold testing 18. We also tested different pacing voltages from 0.5 to 4.2 V, and found that 3 V pulse voltage was the optimal pacing pulse voltage. In the PAMB-G group, we started the pacing voltage at 1 V and reduced by 0.1 V each time until we reached the voltage (0.4 V) where we were unable to pace the heart. Our data showed that the pacing threshold voltage for PAMB-G was less than 1 V and was 3-4-fold lower than that of electrode-only control or gelatin pacing electrodes. Similarly, our *in vivo* A-V block study showed that the threshold for PAMB-G was less than 0.5 V, and was ~3 fold lower than that for the control or gelatin pacing electrodes. Both *ex vivo* and *in vivo* data showed that the pacing threshold voltage was less than the current clinically used voltage of 1.5 V 27, 28. These data suggested that PAMB-G pacing could significantly reduce the threshold voltage, thereby decreasing energy consumption.

It is known that the electrode-myocardial tissue interface plays an important role in cardiac pacing. With external pacing, the current at the electrode tip must generate an electrical field. If the electrical field around the myocardial cells reaches its threshold voltage, voltage-gated sodium channels open on the cell membrane and an action potential is generated 8. As the amount of myocardial fibrosis increases, increased tissue impedance reduces myocardial conductivity and delays electrical signal propagation, contributing to a higher pacing threshold 6. Reducing myocardial tissue impedance can therefore decrease the depolarization threshold. In our equivalent circuit model analysis, injection of PAMB-G hydrogel into the electrode-tissue interface significantly decreased the impedance and increased myocardial cell membrane voltage, facilitating the electrical current energy transfer and thereby enhancing the efficacy of cardiac pacemaker.

There are a few limitations to the study. First, this study did not investigate the inflammatory response of PAMB-G. Thus, the association between fibrosis and the pacing threshold increase remains unanswered. Moreover, we focused on pathophysiological conditions, but less so in analyzing the physics behind the biomaterial's effectiveness. Since the pacing threshold voltage was reduced with conductive biomaterial, the stimulation-induced myocardial fibrosis should be minimal. In addition, our previous study in a rat MI model did not find inflammatory reaction, cellular necrosis, or fibrotic tissue formation at the injection site 14. Second, the current study is an acute evaluation, owing to the limitation of being unable to implant pacemakers in rats for chronic study. This study is used for proof-a-concept evaluation. Future studies will be carried out in large animal models, such as porcine or canine models, for long term study.

## Conclusions

We generated a highly conductive biomaterial, PAMB-G hydrogel. Injection of PAMB-G into the electrode-tissue surface decreased cardiac pacing threshold voltage while maintaining better electrophysiological performance compared with direct electrode or non-conductive biomaterial pacing.

## Supplementary Material

Supplementary figures and tables.

Supplementary movie S1.

Supplementary movie S2.

Supplementary movie S3.

## Abbreviations

AD: adenosine
AMB: 3-amino-4-methoxybenzoic acid
AV: atrioventricular
di-4 ANEPPS: 4-(2-(6-(dibutylamino)-2-naphthalenyl) ethenyl)-1-(3-sulfopropyl)-pyridinium
ECG: electrocardiogram
EDC: N-(3-Dimethylaminopropyl)-N'-ethylcarbodiimide hydrochloride
MEA: multi-electrode array
MS: millisecond
NHS: N-hydroxysuccinimide
PAMB-G: poly-3-amino-4-methoxybenzoic acid-gelatin
