# Supplementary material for: Injectable conductive hydrogel can reduce pacing threshold and enhance efficacy of cardiac pacemaker
Source: Theranostics. 2021 Feb 6;11(8):3948–60. doi: 10.7150/thno.54959 (PMC7914366; doi:10.7150/thno.54959)
Supplement: Supplementary file 1 — Supplementary figures and tables. [file thnov11p3948s1.pdf]

Supplementary Material

for

Injectable conductive hydrogel can reduce pacing threshold and enhance efficacy of cardiac pacemaker

Zhao An[1, 2], Jun Wu[1], Shu-Hong Li[1], Shanglin Chen[1], Fang-Lin Lu[2], Zhi-Yun Xu[2#], Hsing-Wen Sung[3#], Ren-Ke Li[2#]

[1]Toronto General Hospital Research Institute, Division of Cardiovascular Surgery, University Health Network, Toronto, Ontario, Canada

[2]Department of Cardiovascular Surgery, Changhai Hospital, Naval Medical University, Shanghai, China

[3]Department of Chemical Engineering, National Tsing Hua University, Hsinchu, Taiwan (ROC)

Supplementary Figure

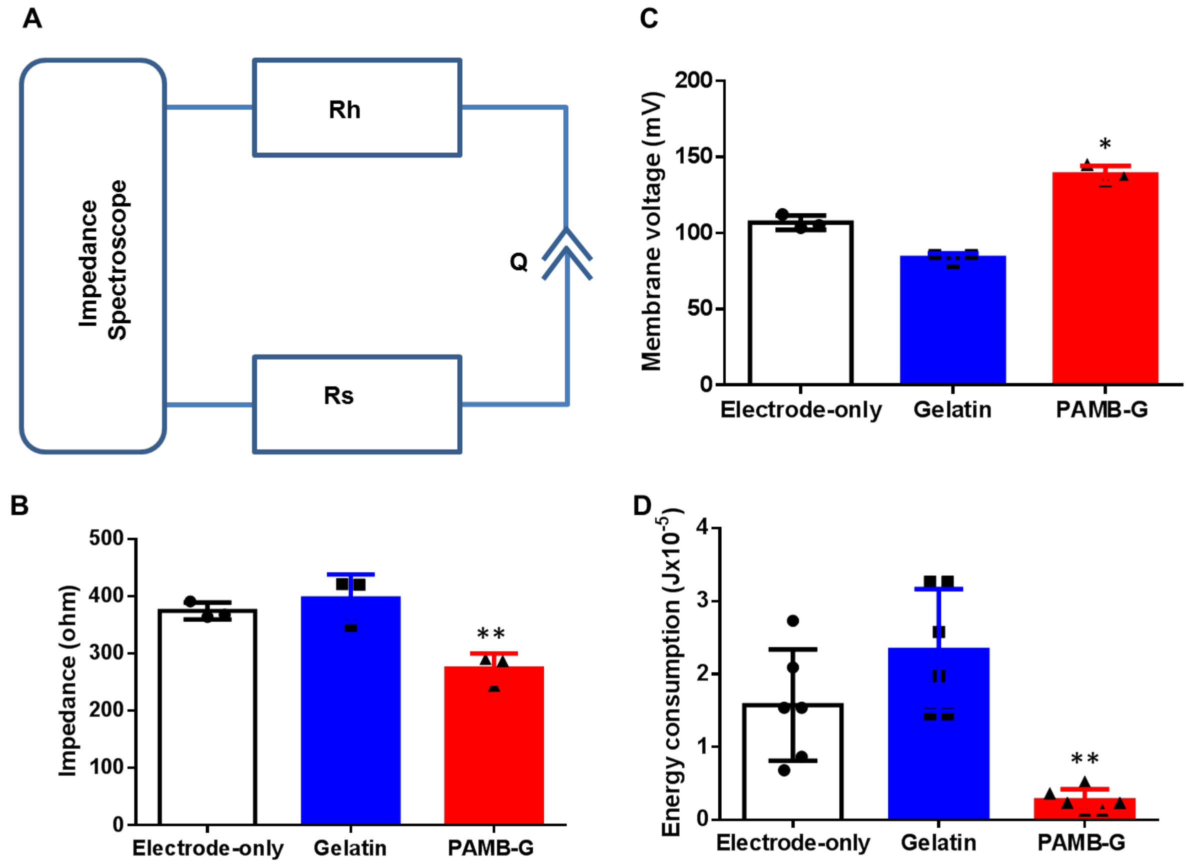

Figure S1: Equivalent circuit model of biomaterials. (A) Schematic drawing of equivalent circuit model. (B) Injection of PAMB-G hydrogel into the electrode-tissue interface significantly decreased myocardial tissue impedance at frequency of 7 kHz compared to the control groups (n = 3/group, **P < 0.01 compared with electrode-only and gelatin). (C) Injecting PAMB-G hydrogel into the electrode-tissue interface significantly increased myocardial cell membrane voltage compared to the control groups (n = 3/group, *P < 0.05 compared with electrode-only and gelatin). (D) Pacing energy consumption was significantly decreased in the PAMB-G group (n = 6/group, **P < 0.01 compared with electrode-only and gelatin). Data analysis used one-way analysis of variance (ANOVA) followed by Tukey's *post-hoc* tests. Data shown as mean ± SD.

Supplementary movies

Movie S1. Under 0.5 V stimulation, the optical mapping movie showed that the stimulation in electrode-only group did not change the autonomous heart rhythm, and a local depolarization in the electrode insert area was detected (small black arrow). Activation orientation was indicated by a large black arrow.

Movie S2. Under 0.5 V stimulation, the optical mapping movie showed a local depolarization in the gelatin injection area (black circle), reflecting the low conductivity of gelatin. The stimulation did not change autonomous heart rhythm. Activation orientation was indicated by a black arrow.

Movie S3. Optical mapping movie showed that 0.5 V stimulation is enough to change the rhythm from autonomous cardiac rhythm to the pacing rhythm, and an ectopic pacemaker at the PAMB-G injected area was detected (small black arrow). Activation orientation was indicated by a large black arrow.
